# Prospects for new leprosy diagnostic tools, a narrative review considering ELISA and PCR assays

**DOI:** 10.1590/0037-8682-0197-2020

**Published:** 2020-11-25

**Authors:** Rafael Silva Gama, Lázaro Azevedo Leite, Lívia Tavares Colombo, Lúcia Alves de Oliveira Fraga

**Affiliations:** 1 Universidade Vale do Rio Doce, Laboratório de Biologia Molecular, Governador Valadares, MG, Brasil.; 2 Universidade Federal de Juiz de Fora, Departamento de Ciências da Vida, Governador Valadares, MG, Brasil.

**Keywords:** Leprosy, Polymerase Chain Reaction, Serologic Tests

## Abstract

Slit skin smear and histopathological examinations are currently the main laboratory tools used to aid the diagnosis of leprosy. However, their sensitivity is low, and many cases are not detected. New methodologies have been studied to develop more accurate tests. This narrative review aims to raise attention to the results of molecular (polymerase chain reaction) and serological (Enzyme-Linked Immunosorbent Assay) tests applied to the diagnosis of leprosy, and to summarize the available information about the former. Original scientific articles published in indexed international journals, whose study involved aspects of the diagnosis and classification of leprosy cases or home contacts, were selected. The data were extracted independently using a standardized method that dictated the inclusion of the following information: diagnosis in Paucibacillary and Multibacillary cases and in household contacts; sample number; sample type; study design; studied variables; statistical analysis employed; main results; and limitations identified. In clinical practice, the results from molecular and serological tests are assessed separately, with moderate sensitivity and specificity. However, an integrated study of these methodologies has been suggested for greater accuracy in diagnosis.

## INTRODUCTION

Leprosy is a chronic granulomatous infection that mainly affects the skin and peripheral nerves, presenting several clinical manifestations due to the pattern of the immune response established as a result of infection with *Mycobacterium leprae* ^1^. The disease is considered a public health issue in countries where the annual prevalence rate is greater than 1 case per 10,000 inhabitants. It is endemic in several countries with low levels of social and economic development, especially India and Brazil, with the highest absolute number of cases in the former. In 2018, 208,619 new leprosy cases were registered worldwide by the World Health Organization (WHO). Preliminary data for 2019 show 120,334 and 26,612 new leprosy cases for India and Brazil, respectively, both classified as high-load countries^2^ ^,^ ^3^.

Transmission likely occurs via the upper respiratory airway through prolonged contact with untreated infected patients. Poor socioeconomic and sanitary conditions as well as genetic predisposition, seem to play an essential role in the development of the disease^4^ ^,^ ^5^.

Early detection of the disease is a strategy to stop the transmission of *M. leprae* and to prevent the occurrence of physical disability. However, leprosy is still mainly diagnosed based on clinical examination, and in many cases the symptoms are subtle and often not noticed by specialists. Slit skin smear and histopathological examinations are used to aid the clinical diagnosis and are useful in spectral and treatment categorization^6^. New methodologies have been studied for the development of more effective tests. Among these, molecular and serological assays such as polymerase chain reaction (PCR), Enzyme-Linked Immunosorbent Assay (ELISA), and rapid tests have stood out. This narrative review aims to raise awareness of the results of molecular and serological tests applied to the diagnosis of leprosy and to highlight key points about the former.

## CURRENT DIAGNOSTIC ASSAYS

The bacilloscopic exam evaluates the bacillary load and morphology. In Brazil, the Ministry of Health recommends performing a slit skin smear from four sites. The preferred sites are active lesions or lesions with altered sensitivity as well as the ear lobes and the contralateral elbow. In the absence of injury, intradermal shaving should be performed on both ear lobes and elbows^7^. The slit skin smear exam has a specificity close to 100%; however, it has low sensitivity, since it is positive in only 30% of infected patients^8^. Patients with up to five skin lesions are considered paucibacillary (PB), and those with six or more lesions are classified as multibacillary (MB). A slit skin smear should be performed, if available, and a positive result classifies the case as MB. However, a negative result does not rule out the clinical diagnosis of leprosy and does not necessarily classify the patient as PB^9^.

Histopathological examination is often performed to confirm clinically dubious cases. Additionally, it is used as one of the criteria in the Ridley-Jopling spectral classification that defines five spectral types of leprosy. The predominant ones are the polar tuberculoid (TT) and the lepromatous (LL) forms. The TT form has a low bacillary load, high cellular immune response, and low antibody production, while the LL form has a high bacillary load, increased antibody production, and reduced cellular immune response. In addition to these, immunologically and clinically unstable forms are described, including borderline-tuberculoid (BT), borderline-borderline (BB), and borderline-lepromatous (BL)^10^. Early diagnosis of leprosy in subclinical infections could be essential for the rapid interruption of the disease transmission chain, and to prevent the development of leprosy sequelae by prompt treatment. Therefore, the establishment of a sensitive test for leprosy diagnosis has been a leading research objective^11^.

## MOLECULAR ASSAYS

Unlike slit skin smears, which require about 10^4^ ^)^ bacilli per gram of tissue for reliable detection, polymerase chain reaction (PCR) is a technique with high specificity and sensitivity, capable of detecting 25 fg (10^-15^ g) of deoxyribonucleic acid (DNA) from *M. leprae*. Moreover, the possibility of its use in almost all types of clinical samples gives this method a high potential for differential diagnosis^12^ ^,^ ^13^. Different sequences and target genes have been used for the amplification of *M. leprae* DNA, mostly from skin biopsies, by PCR. Specific and straightforward systems were used to amplify gene regions that encode the *M. leprae* 36-kDa^14^ ^,^ ^15^ or 65-kDa^16^ ^)^ antigens, repetitive sequences (RLEP), and the 16S ribosomal RNA (rRNA) subunit^17^ ^,^ ^18^ ^,^ ^19^. 

To develop a more sensitive and specific method for the molecular detection of *M. leprae*, was compared the PCR amplification of two regions, the 18-kDa gene and the RLEP, which was more sensitive^12^. The relatively high number of copies of the RLEP sequence in the *M. leprae* genome, estimated to contain at least 28 copies, likely gives this target a greater sensitivity compared to single-copy genes^17^. Goulart and Goulart (2008)^13^ showed that among several nucleic acid markers for the diagnosis of leprosy, three had greater sensitivity and specificity (RLEP, Ag 85B, and 16S rRNA). Later, Martinez et al. (2011)^18^ demonstrated that the quantitative real-time PCR assay (qPCR) for the RLEP sequence could be used to improve the detection of infection in MB patients, due to its high sensitivity (100%) and specificity (84.6%). The authors concluded that qPCR positivity may indicate the presence of bacilli or subclinical infections, which does not mean that the condition will progress to disease. However, the RLEP is highly conserved in bacteria of the same genus, which raises some questions about its usage as a diagnostic marker.

A study by Martinez et al. (2011)^18^ compared the sensitivity and specificity of qPCR in the amplification of the sodA, 16S rRNA, RLEP, and Ag 85B genes for the differential diagnosis of leprosy, confirming that the RLEP sequence confers greater sensitivity to the technique. However, the RLEP sequence was amplified in four patients with other skin diseases. In comparison, the amplification of the 16S rRNA gene, although less sensitive, was specific for *M. leprae*. In a previous study, Martinez et al. (2009)^20^ confirmed the specificity of the *M. leprae* 16S rRNA qPCR primers by testing them with 9 other *Mycobacterium* species. Gama et al. (2018)^21^, using qPCR to detect *M. leprae* DNA in earlobe dermal shavings and blood samples from leprosy cases and household contacts living in an endemic area, identified the infection in 23.89% of clinically asymptomatic contacts. These data corroborate the high potential of this tool for the early diagnosis of leprosy.

## SEROLOGICAL ASSAYS

Serological tests are aimed at detecting specific antibodies against *M. leprae* that indicate infection. These tests can be useful in monitoring the effectiveness of therapy, determining the prevalence of the disease, and assessing the distribution of infection in a particular community^22^ ^,^ ^23^. The elucidation of the chemical structure of Phenolic glycolipid 1 (PGL-I), a specific antigen of *M. leprae*, in 1981 made it possible to create serological tests for diagnosis^24^. Studies involving PGL-I mainly use the ELISA technique^25^. The low cost and quantitative results have made anti-PGL-I serology a widely used method. In addition to ELISA, rapid anti-PGL-I assays have been developed, such as the Dipstick^22^ and lateral flow immunochromatographic assays including the ML Flow^26^ and ML ICA. These are more straightforward tests than ELISA, as they do not require the use of laboratory equipment or a specialized laboratory technician, and provide reproducible results^27^. Anti-PGL-I serology can identify patients for early monitoring and treatment and can reduce neural damage and disability^28^.

After the *M. leprae* genome was published^29^, new bacillus-specific proteins or peptides with potential applicability in the diagnosis of leprosy were identified^30^ ^-^ ^37^. Serology results using recombinant *M. leprae* proteins reflect the immune spectrum of the disease: high levels of antibodies at the lepromatous pole and lower levels of antibodies at the tuberculoid pole^32^. In a study that evaluated the humoral and cellular immune response to 33 *M. leprae* recombinant proteins, three (ML0405, ML2055, and ML2331) were identified as immunogenically capable of inducing a specific cellular immune response in PB patients and humoral response by production of IgG in MB patients. From these advances, Leprosy IDRI Diagnostic-1 (LID-1) was obtained by fusing the ML0405 and ML2331 genes to produce a single chimeric protein with better sensitivity^31^ ^,^ ^38^.

Subsequently, the LID-1 and PGL-I epitopes were conjugated to form NDO-LID, ensuring the immunoreactivity of the two isolated proteins, indicating potential application in serological diagnosis, mainly in the early detection of cases^39^ ^,^ ^40^. According to Frade et al. (2017)^41^, the commercial rapid test NDO-LID (Orange Life, Rio de Janeiro, Brazil) was positive in 62.8% of patients clinically diagnosed with leprosy. However, it showed less specificity than the anti-PGL-I and anti-LID-1 ELISAs. Although this test can identify dominant responses to both the glycolipid (IgM anti-PGL-I) and protein (IgG anti-LID-1), the NDO-LID has the same limitation as other rapid diagnostic tests, highlighting the difficulty of using this test to monitor individuals in the early stages of the disease and/or PB. Regardless, the use of serological tests associated with clinical examination, can contribute to the early detection and treatment of leprosy. Serological tests perform better in the identification of MB patients, especially the BL and LL forms. Additionally, BL and LL patients produce high IgM titers against PGL-I, while TT patients have low levels of specific antibodies^23^.

## CONTACT SURVEILLANCE: IMPORTANCE OF MOLECULAR AND SEROLOGICAL TESTS

Contact surveillance is done using a dermatoneurological exam. The aim of contact surveillance is to diagnose new cases among people who have prolonged close interactions with a newly diagnosed leprosy patient (called an index case). Contacts of leprosy cases have a higher risk of illness since they have a greater exposure to M. leprae than the general population^42^. Two groups of people are considered close contacts, those who reside or used to reside with the patient (household contacts) and those who interact or used to interact closely with the patient (social contacts)^9^.

The detection of *M. leprae* DNA has been carried out using several clinical samples, such as nasal swabs, urine, saliva, and skin biopsies^43^ ^,^ ^44^ ^,^ ^45^. In household contacts, the detection of *M. leprae* DNA by PCR of nasal swabs does not determine whether the contact will progress to active disease. However, PCR is a very specific test, and several specialists advise treatment of all PCR-positive contacts. The rates of DNA detection in nasal swabs from contacts vary from 1% to 10% depending on the clinical form of the index case. The positivity rates observed among healthy individuals raise questions about the feasibility of using PCR on nasal swab samples to predict the risk of developing the disease. Recently, studies have indicated that the risk of progressing to active disease increases if the contact is positive for blood PCR^46^ ^,^ ^47^. 

Anti-PGL-I seropositivity and the hidden prevalence of leprosy among household contacts and school-aged children indicate the presence of active infection foci. The serological test can be used to identify school-age children with a higher risk of developing leprosy^48^. The high prevalence of PGL-I seropositivity among contacts of MB patients shows that subclinical infection might be common^49^ ^-^ ^51^. The detection of antibodies against PGL-I identifying infected contacts without apparent clinical signs can be an auxiliary tool for leprosy control programs.

The reactivity of IgM and IgG antibodies against NDO-LID may allow the detection of infections at an early stage^40^. Additionally, household contacts with anti-PGL-I^31^ ^,^ ^52^ ^,^ ^54^and anti-NDO-LID^39^ ^,^ ^53^ seropositivity have a higher risk of developing leprosy^31^ ^,^ ^39^ ^,^ ^52^ ^-^ ^54^. The successive analysis of antibody reactivity can be useful, since the increase in anti-PGL-I and anti-LID-1 titers could identify household contacts that require further monitoring or be an indicator for conducting a clinical examination^13^ ^,^ ^37^ ^,^ ^44^.

Household contacts, in addition to having a subclinical infection, may also be actively involved in the spread of *M. leprae* to susceptible individuals in endemic regions, a worrying factor, since they may contribute to the leprosy transmission chain^44^.

In areas of greater endemicity, serological and molecular tests have been carried out and analyzed separately. However, Gama et al. (2019)^55^ proposed the integration of these methods to assist in the diagnosis and monitoring of household contacts using the Random Forest algorithm for data analysis. This is a robust multivariate analysis, able to build classification trees with minimal error rates, which is favorable for the diagnosis of diseases such as leprosy^56^ ^,^ ^57^.

Gama et al. (2019)^55^, used the classification model “Sick × Healthy” in the Random Forest analysis based on serological, molecular, and bacilloscopic data, to evaluate the prediction of infection in clinically diagnosed cases and household contacts, accompanying them for five years. This model, Random Forest, highlighted the possibility of early diagnosis of MB (90.5%) and especially PB (70.6%) cases, while separately evaluated tests did not reach the same high rate of diagnostic correctness.

## DISCUSSION

## PERSPECTIVES FOR NEW LEPROSY DIAGNOSTIC TOOLS

Early detection of leprosy is a strategy to interrupt the transmission of *M. leprae* and to prevent the occurrence of physical disability, a serious consequence. However, the diagnosis is still essentially defined by clinical examination. Slit skin smear and histopathology examinations are used to aid the clinical diagnosis and are useful in spectral and treatment categorization^58^.

A study carried out in a hyperendemic area of Brazil indicated that the anti-LID-1 assay has a sensitivity of 89% and a specificity of 42% for the diagnosis of leprosy. The low specificity is probably related to the presence of a large number of asymptomatic individuals infected with *Mycobacterium* ^59^. Conversely, NDO-LID has a specificity of 85.89% and a sensitivity of 90.6% for MB and 27% for PB^53^. In that study, the authors correlated the ELISA results with the bacteriological index and the Ridley-Jopling classification since the lepromatous pole patients had higher responses. In contrast, in those of the tuberculoid pole, the antibody levels were lower. Other authors confirmed these results^60^ ^,^ ^61^. Additionally, the cases with high bacilloscopy index (BI) have high titers of anti-PGL-1 IgM^41^ ^,^ ^61^ ^,^ ^62^, anti-LID-1 IgG^60^, and anti-NDO-LID IgM and IgG^40^ ^,^ ^53^.

The detection of anti-PGL-I IgM contributes to the correct classification of leprosy cases^27^ ^,^ ^62^ ^-^ ^64^ and helps to differentiate between PB and MB cases^27^ ^,^ ^39^ ^,^ ^40^ ^,^ ^45^ ^,^ ^61^ ^,^ ^65^ ^-^ ^67^. The analysis of IgG reactivity against LID-1 is also able to assist the diagnosis and operational classification of MB leprosy^38^ ^,^ ^40^ ^,^ ^60^ ^,^ ^68^, and IgM and IgG against NDO-LID allow for the rapid and consistent detection of MB leprosy as well as for the monitoring of these cases^39^ ^,^ ^40^.

The reactivity to LID-1 allows the diagnosis of leprosy 6 to 8 months before the clinical diagnosis of the disease. Thus, screening for anti-LID-1 antibodies, either in the population or in groups at risk, can significantly accelerate the treatment of leprosy cases and reduce transmission rates by decreasing the number of individuals who are discharging bacterial load^31^.

It is important to note that serological tests already help determine the most appropriate type of multidrug therapy (MDT)^61^ ^,^ ^62^ ^,^ ^69^ ^,^ ^70^ and contribute to the reduction of possible nerve damage and the evolution of physical disabilities^61^. Additionally, they can help with treatment-related decision-making when smear microscopy is not available, as there is good agreement between serology and BI^66^ ^,^ ^69^ ^,^ ^70^ ^,^ ^71^ ^,^ ^72^. 

Furthermore, the high sensitivity of qPCR to sputum smear microscopy makes this technique extremely important in clinical support for diagnosis^12^ ^,^ ^13^ ^,^ ^20^.

Knowing that leprosy is a disease with a long incubation period and that the symptoms are difficult to perceive in the initial stage of the infection, highlights that the monitoring of contacts with positive qPCR results is extremely important. About 6% to 8% of household contacts develop clinical symptoms of leprosy within two years of the diagnosis of the index case^73^.

Another important factor is the level of exposure of household contacts to the bacillus. Virchowian (VV) index cases transmit *M. leprae* from the nasal mucosa and lesions before starting treatment with polychemotherapy. Therefore, their household contacts are exposed to a higher bacillary burden. Individuals who live with PB index cases have a lower risk of developing leprosy due to the low potential for transmissibility^74^. Moreover, Banerjee et al. (2010)^75^ showed that 10.9% of VV case contacts had positive PCR results and 1.8% of these individuals developed leprosy over 2 years.

On the other hand, an explanation for the negative qPCR results in some patients, especially paucibacillary patients, is the possibility of inhibited amplification of *M. leprae* DNA due to the high concentration of human genomic DNA in the samples, as described by Martinez et al. (2006)^43^.

A way of improving laboratory diagnostic capacity was assessed by the integrated analysis of serological and molecular tests using the Random Forest algorithm, which showed greater sensitivity and specificity in identifying MB and PB cases as well as subclinical infections in household contacts^55^.

Bacillus calmette guerin (BCG) vaccination promotes a certain rate of protection against the development of leprosy, in the general population and in household contacts, especially when applied in two doses^76^. Sales et al*.* (2011)^77^ observed a protection rate of 56% in contacts who received the BCG vaccine. Despite this protection, some contacts developed leprosy, of which 89% presented the PB form, which indicates the protective effect of the vaccine against the development of the VV form.

In addition to BCG vaccination, chemoprophylaxis of contacts has also been evaluated as a strategy for leprosy control. A study by Moet et al. (2008)^76^ in Bangladesh indicated that a single chemoprophylactic dose of rifampicin administered to contacts was effective in preventing the development of leprosy in 57% of treated individuals. This protective effect of chemoprophylaxis was observed for a two-year follow-up period.

Fischer et al. (2011)^78^ used a microsimulation model to compare results from different hypothetical leprosy intervention programs, showing that the strategy of early diagnosis of subclinical infection associated with treatment achieves a higher cure rate compared to the application of chemoprophylaxis alone.

## CONCLUSION

Molecular and serological assays are very promising for the diagnosis of leprosy. Although many studies have been carried out in isolation, the integrated assessment of the methods could increase the sensitivity and specificity, contributing to early diagnosis or monitoring of household contacts, thus promoting greater control of the disease.

The evaluation of protocols and proposal of an integrated diagnostic model of molecular, serological, and clinical techniques for large-scale application is suggested. Additionally, an economic/financial study is essential to enable its implementation in health services. Therefore, we propose the development of prototypes of low-cost molecular and serological tests to implement in central laboratories, such as the Central Public Health Laboratories (LACEN) in Brazil. Clinical data must be associated with laboratory results to validate an integrated analysis model using software or an application to predict leprosy diagnosis.
